# European aquatic ecological assessment methods: A critical review of their sensitivity to key pressures

**DOI:** 10.1016/j.scitotenv.2020.140075

**Published:** 2020-10-20

**Authors:** Sandra Poikane, Fuensanta Salas Herrero, Martyn G. Kelly, Angel Borja, Sebastian Birk, Wouter van de Bund

**Affiliations:** a European Commission Joint Research Centre (JRC), via Fermi 2749, Ispra 21027, Italy; b Bowburn Consultancy, 11 Monteigne Drive, Bowburn, Durham DH6 5QB, United Kingdom; c School of Geography, Nottingham University, Nottingham NG7 2RD, United Kingdom; d AZTI, Marine Research, Basque Research and Technology Alliance (BRTA), Herrera Kaia Portualdea s/n, 20100 Pasaia, Spain; e Department of Aquatic Ecology, Faculty of Biology, University of Duisburg-Essen, 45141 Essen, Germany

**Keywords:** Bioassessment, Ecological status, Biological metrics, Aquatic ecosystems, Water framework directive, Anthropogenic pressures, BQE, biological quality element, WFD, water framework directive

## Abstract

The European Union has embarked on a policy which aims to achieve good ecological status in all surface waters (i.e. rivers, lakes, transitional and coastal waters). In theory, ecological status assessment methods should address the effects of all relevant human pressures. In this study, we analyze the degree to which methods European countries use to assess ecological status tackle various pressures affecting European waters.

Nutrient pollution is by far the best-covered pressure for all four water categories. Out of total of 423 assessment methods, 370 assess eutrophication and pressure-specific relationships have been demonstrated for 212 of these. “General degradation” is addressed by 238 methods, mostly validated by relationships to combined pressure indices.

Other major pressures have received significantly less effort: hydromorphological degradation is assessed by 160 methods and pressure-specific relationships have been demonstrated for just 40 of these. Hydromorphological pressures are addressed (at least by one BQE) only by 25% countries for coastal waters and 70–80% for lakes and transitional waters. Specific diagnostic tools (i.e. single-pressure relationships) for hydromorphology have only been developed by a few countries: only 20% countries have such methods for lakes, coastal and transitional waters and less than half for rivers. Toxic contamination is addressed by 90 methods; however, pressure-specific relationships have been demonstrated for just eight of these. Only two countries have demonstrated pressure-specific acidification methods for rivers, and three for lakes.

In summary, methods currently in use mostly address eutrophication and/or general degradation, but there is not much evidence that they reliably pick up the effects of other significant pressures such as hydromorphology or toxic contamination. Therefore, we recommend that countries re-examine: (1) those pressures which affect different water categories in the country; (2) relevant assessment methods to tackle those pressures; (3) whether pressure-response relationships have been developed for each of these.

## Highlights

• Large number of aquatic biological assessment methods (423) used in EU member states
• Not all major pressures are addressed by biological assessment systems.
• Eutrophication is best-covered (370 methods) with pressure-response well documented.
• Lack of assessment systems addressing hydromorphological alterations and toxic contamination
• The capacity of assessment systems to diagnose the cause of degradation is limited.

## Graphical abstract

**Figure:**
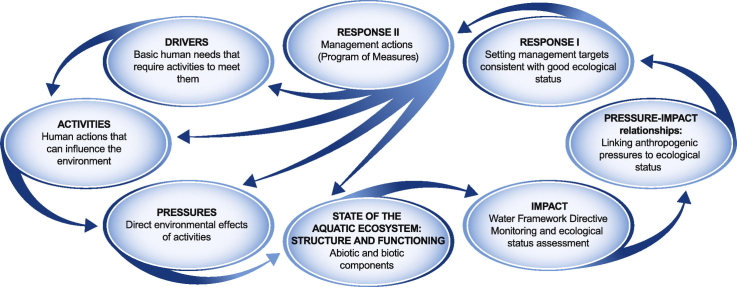

## 1 Introduction

In Europe, the United States, Australia and China, assessment of ecological status (= ecological integrity, ecosystem health) has become a benchmark for both scientific and management purposes (Borja et al., 2008; Karr and Chu, 2000; Xu et al., 2014). In many studies, ecological assessment outcomes are considered as a basis for objective comparisons among countries and over time (EEA [European Environment Agency], 2018; Grizzetti et al., 2017). However, the reality is more nuanced, as ecological status depends on how assessment methods are designed and used (Langhans et al., 2014; Probst and Lynam, 2016). The essential steps include field sampling and processing, calculation of biological metrics, their combination into final assessment and classification: all these can be done in countless different ways (Birk et al., 2012). Moreover, ecological assessment can be built as “pressure-specific” (targeting a single human pressure) or “multi-pressure” (addressing a range of pressures) (Hering et al., 2006).

For instance, pressure-specific methods have been developed in Europe for acidification (lakes: McFarland et al., 2010; rivers: Juggins et al., 2016), hydromorphological degradation (lakes: Urbanič, 2014; rivers: Lorenz et al., 2004), eutrophication (lakes: Carvalho et al., 2013; rivers: Kelly et al., 2008; coastal waters: Vollenweider et al., 1998), organic pollution (rivers: Brabec et al., 2004; coastal waters: Word, 1980) and toxic pollution (rivers: Archaimbault et al., 2010).

However, most of the assessment methods address multiple pressures (e.g., lakes: Poikane et al., 2017; rivers: Mondy et al., 2012; coastal and transitional: Borja et al., 2015, Borja et al., 2019). In some cases, the meaning of “a range of pressures” has been disentangled, e.g. for lakes: eutrophication and hydromorphological alterations (several benthic invertebrate assessment methods; Poikane et al., 2016); for rivers: nutrients, organic and toxic pollution and habitat degradation (French benthic invertebrate assessment method; Mondy et al., 2012); for transitional waters: nutrients, organic matter, hazardous substances and fishing pressure (Polish fish-based assessment system; Smoliński and Całkiewicz, 2015); for coastal waters: nutrients, organic matter, hydromorphological alterations and hazardous substances (POMI index for *Posidonia oceanica* seagrass; Romero et al., 2007). However, many methods address “general degradation” and it is not always clear which pressures are included and which management measures are needed to improve the ecological status (Carvalho et al., 2019; Lemm et al., 2019).

In Europe, the main legislation to achieve good ecological status in surface waters through management of human-derived pressures is the Water Framework Directive (WFD; European Commission, 2000). To fulfil the requirements of this, EU member states have to develop biological assessment methods using a range of Biological Quality Elements (BQEs) (i.e. phytoplankton, benthic flora, benthic invertebrates, and fish fauna) and regulatory thresholds have to be compared and harmonized among countries (Poikane et al., 2014). Surface waters are affected by multiple pressures, which can interact in additive, synergistic or antagonistic ways (Schinegger et al., 2012). Their impact might be exacerbated (or in some cases mitigated) by climate change (Paerl et al., 2019). In addition, new pressures are emerging (e.g. microplastic pollution, pharmaceuticals, light and noise, freshwater salinization; Reid et al., 2019). Therefore, general assessment methods have a crucial role in informing and guiding water policy practices. However, more frequently, these do not discriminate between different types of pressure, so diagnostic tools are needed to identify the cause(s) of ecological status degradation and to guide the choice of relevant management measures (Baattrup-Pedersen et al., 2019). Such tools should be pressure-specific, with demonstrated pressure-response relationships (e.g., eutrophication: Carvalho et al., 2013; Poikane et al., 2019; acidification: McFarland et al., 2010; hydromorphological alteration: Urbanič, 2014). In some cases, pressure-specific modules or metrics have been developed within multi-pressure methods (Baattrup-Pedersen et al., 2019; Böhmer et al., 2004), forming a system capable of distinguishing between different impacts on the ecological status; however, such examples are rare.

**Fig. 1:**
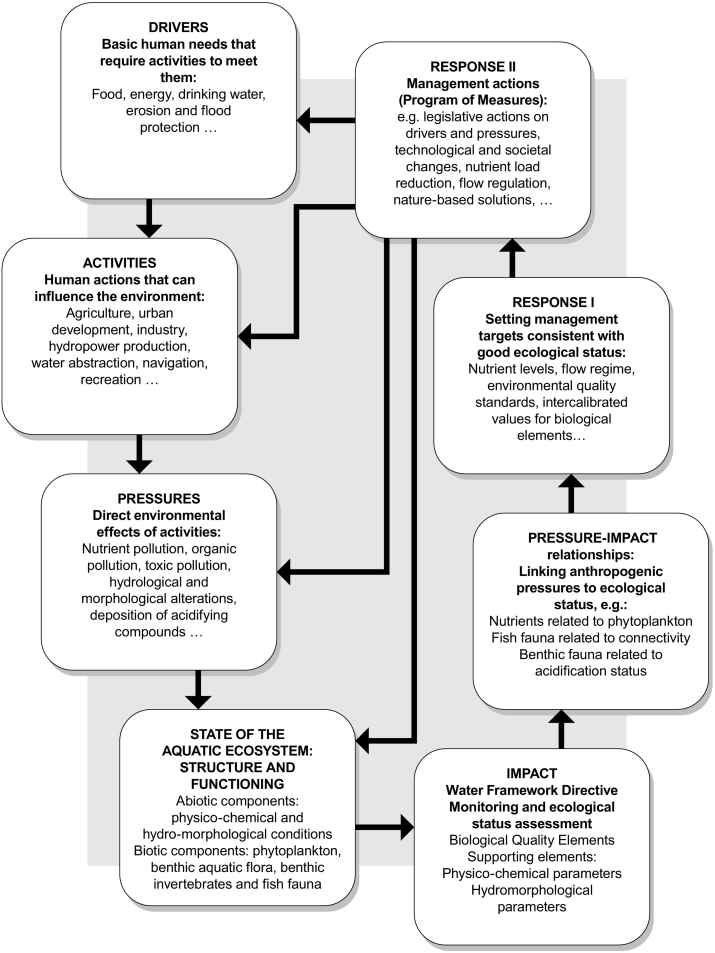
Conceptual scheme of the links between drivers, activities and pressures to ecosystem structure, functioning, and the related impacts assessment, under the Water Framework Directive (based on Solimini et al., 2009; Elliott et al., 2017; Anzaldua et al., 2018; EEA [European Environment Agency], 2018).

Pressure-response relationships link ecological status to pressures, which allows management targets to be set and restoration measures to be devised (Hering et al., 2006; Karr and Chu, 2000). Historically, these relationships have been studied under the DPSIR (Drivers-Pressures-State-Impacts-Responses) framework (Patrício et al., 2016). In recent times, this framework has been extended by adding human Activities, Welfare (as a proxy of the benefits from ecosystem services) and Measures, to complete the DAPSI(*W*)R(M) framework (Elliott et al., 2017). Based on this extended framework, the concept followed in this study integrates the WFD perspective to show the links between drivers and activities that can result in pressures to the environment (Fig. 1). The pressures can change the state of the aquatic environment (both in abiotic and biotic elements of the WFD), resulting in impacts of those elements, which must be assessed using different methods (Birk et al., 2012). The WFD requires the need to link the pressures and impacts when assessing the ecological status to allow for devising an adequate management response. This response is built on harmonized targets of good ecological status (intercalibrated and published by EC, 2018) and formalized in national programs of measures that each EU member state must develop to reduce/remove pressures and achieve good ecological status (Fig. 1).

Developing pressure-response relationships is, for many reasons, not an easy task and has been tackled in many different ways, as summarized in in the following:

• In many cases, pressure-specific relationships have been validated against relevant pressure indicators, for instance, nutrients for eutrophication (Lyche Solheim et al., 2013), organic matter content in the sediment for organic enrichment (Subida et al., 2012), pH for acidification (Poikane et al., 2016) and specific metrics/indices for hydromorphological pressures (Lorenz et al., 2004).
• For multi-pressure methods, the relationship to pressures has been demonstrated mainly with combined pressure indices (Harrison and Kelly, 2013; Poikane et al., 2016, Poikane et al., 2017), multiple regressions or more complex statistical procedures (Breine et al., 2015; Mondy et al., 2012).
• In many other cases, relationships have been validated only against catchment land use (Kuhar et al., 2011; Pinedo et al., 2015), or have not been validated at all (e.g., Padisak et al., 2006).

The absence of a link between assessment and management is considered as a major flaw of the WFD implementation process, hindering achievement of good ecological status (Carvalho et al., 2019). In addition, concerns have been raised that not all pressures are addressed adequately by the assessment methods currently in use (Carvalho et al., 2019; Lyche Solheim et al., 2013; Reyjol et al., 2014). So far, the primary focus has been on developing assessment methods for all BQEs prescribed in the WFD. However, it is equally important that all relevant major pressures can be diagnosed in order to guide the choice of management measures. Developing methods for all BQEs does not necessarily mean that all relevant major pressures are addressed. For example, in lakes not only phytoplankton, macrophytes and phytobenthos (Kelly et al., 2016), but also benthic invertebrates and fish (Lyche Solheim et al., 2013; Poikane et al., 2016) are used to assess eutrophication, whilst acidification and hydromorphological pressures in lakes are largely ignored (Poikane et al., 2020). The lack of specific assessment methods might not be important for pressures having similar and closely related effects (e.g. nutrient and organic pollution); however, it is possible that pressures such as acidification and toxic pollution might be missed by methods designed to assess eutrophication.

Here, we argue that (1) all major pressures present in a particular country should be addressed by assessment methods and (2) diagnostic tools (e.g. single-pressure relationships) should be developed for at least one BQE for each of these pressures to provide an informed choice of management measure(s). Despite the importance of these issues, no overview or analysis has been conducted to date, which pressures are addressed or missing by biological assessment methods in Europe and whether pressure-specific relationships are available for management planning.

In this study, we first investigated which pressures are assessed by ecological assessment methods currently in use in EU member states, and then explored whether or not pressure-response relationships have been demonstrated. Further, we assess the extent to which existing pressures might remain undetected/undiagnosed by biological assessment methods and provide recommendations for future developments.

## 2 Material and methods

### 2.1 Data

The data used for this exercise were collated within the official reporting procedure of the WFD intercalibration exercise (Poikane et al., 2014). For each national assessment method, the following information has been reported: (1) detailed description of method, including sampling, data processing, metrics and boundary setting procedure; (2) pressures addressed; (3) pressure-response relationships, including the strength of the relationships (correlation coefficients or coefficients of determination, along with statistical significance *p*); and (4) scientific and technical literature supporting the method. The information has been collated in a database, including all methods reported in EC, (2018) along with a few intercalibrated subsequently (until April 2019). Data gaps have been filled by consulting scientific and technical references supporting assessment methods. All methods are listed in Supplementary Data Tables S1-S4.

### 2.2 Pressure categories

The main pressures reported to be assessed by countries include: (1) eutrophication; (2) organic pollution; (3) acidification; (4) hydromorphological alteration; (5) toxic pollution; (6) aquatic invasive species; and (7) “general degradation”. Many different types of hydromorphological alteration have been reported, including connectivity loss, habitat destruction, water level fluctuations, riparian habitat alterations, impoundments and hydropeaking; all these alterations have been included in the “hydromorphology” group for the sake of simplicity. Similarly, different types of toxic contaminants (e.g., heavy metals, organochlorine pesticides) have been combined in a single “toxic pollution” category. Finally, the pressure reported as “land use changes” (which is actually a driver according to the DPSIR) has been merged with “general degradation”.

### 2.3 Categories of pressure-impact relationships

Information on pressure-impact relationships was categorized as follows (Table 1):

(A) Relationship to single pressure (regression against a proxy for pressure; e.g. phosphorus or nitrogen concentration for eutrophication; biochemical oxygen demand (BOD) and sediment organic content for organic pollution; pH for acidification; a morphological index for hydromorphological alterations, etc.).
(B) Relationship to abiotic multi-pressure index combining two or more pressures – such indices have been widely used for assessment methods in rivers (Logez and Pont, 2011), lakes (Poikane et al., 2017), coastal (Korpinen et al., 2012), and transitional waters (Lepage et al., 2016).
(C) Relationship to biological common metrics used for intercalibration (Intercalibration Common Metric; ICM) responding to two or more pressures. Such indices were especially widely used for lakes and rivers: lake benthic invertebrates (Poikane et al., 2016), river benthic invertebrates (Erba et al., 2009), river fish (Segurado et al., 2014), river and lake phytobenthos (Kelly et al., 2014).
(D) Relationship not sufficiently demonstrated (no regression or only weak regressions, i.e. *R* < 0.3; Cohen, 2013).

**Table 1:** Categorization of pressure-impact relationships.

Category	Pressure-impact relationship	Interpretation	
A	Relationship to single pressure	Pressure-specific relationship	Evidence provided
B	Relationship to pressure index (two or more pressures)	Multi-pressure relationship	
C	Relationship to intercalibration common metric (responding to two and more pressures)		
D	Not sufficiently demonstrated *R* < 0.3	Not demonstrated	No evidence provided

The level of proof is, therefore, a country'sc own demonstration of a significant statistical association with one or more pressures. Readers should be aware that the absence of evidence for relationships with other variables may reflect study design (i.e. omission of potential stressors from a dataset) rather than conclusive evidence of the absence of an effect.For interpretation, methods were grouped into three categories: pressure-specific relationships (Category A), multi-pressure relationships (Categories B and C) and not demonstrated (Category D).

Data on the strength of reported pressure-response relationships was collated (expressed as coefficients of determination (r^2^^)^; correlation coefficients were converted into r^2^). Only significant (*p* < .05) relationships with r^2^ > 0.1 were included in the analysis. Statistical analyses of reported threshold values were performed using R 3.5.3 (R Core Team, 2019). The significance of different criteria setting methods was tested by Kruskal-Wallis Rank Sum Test and post-hoc Dunn's Test (Dinno, 2015). Effects were considered statistically significant at *p* < .05.

## 3 Results

### 3.1 Overview of biological assessment methods and pressures assessed

In total, 423 methods from 29 countries^1^ were included in this analysis: 141 for rivers, 107 for lakes, 109 for coastal waters and 66 for transitional waters. Methods for benthic flora were most numerous (*n* = 173 methods), owing to the fact that countries have developed separate methods for macrophytes and phytobenthos (inland waters, *n* = 96) and angiosperms and macroalgae (coastal and transitional waters, *n* = 77). The next most common BQE was benthic invertebrates (*n* = 113), followed by phytoplankton (*n* = 78) and fish (*n* = 59), as phytoplankton is not used for river assessment (except very large rivers) and fish are not used for coastal waters.

**Fig. 2:**
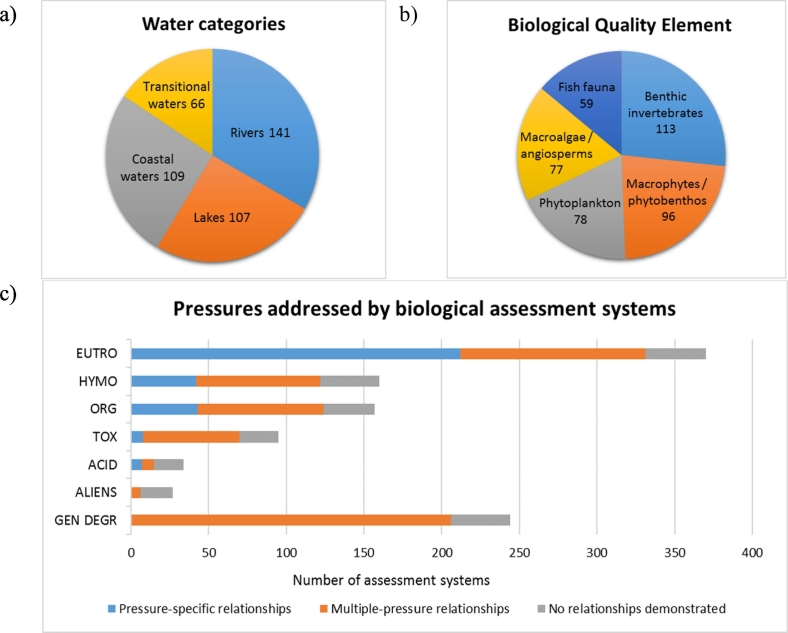
Overview of aquatic assessment methods for the Water Framework Directive (WFD); a) water categories; b) biological quality elements (BQE); c) pressures assessed and relationships demonstrated. EUTRO: eutrophication; HYMO: hydromorphology; ORG POLL: organic pollution; TOX: toxicity; ACID: acidification; GEN DEGR: general degradation.

Most of the methods address eutrophication (*n* = 370) and “general degradation” (*n* = 244), pressures less addressed are hydromorphology (*n* = 160), organic pollution (*n* = 157), toxic pollution (*n* = 95) and acidification (*n* = 34) (Fig. 2). Few methods address only one pressure for rivers (n = 9), usually eutrophication or acidification, while most of the methods address two or more pressures (*n* = 132 for rivers). However, for other water categories the number of pressure-specific methods is higher (*n* = 42 for lakes, *n* = 44 for coastal watersal, *n* = 23 for transitional waters) and forms a significant part (35–40%) of all methods.

Pressure-response relationships are demonstrated in around 80% of cases (although only 29% have pressure-specific relationships); eutrophication is most commonly addressed by pressure-specific relationships (57%), followed by organic pollution (27%) and hydromorphology (26%).

### 3.2 Pressure-response relationships demonstrated by water category

**Fig. 3:**
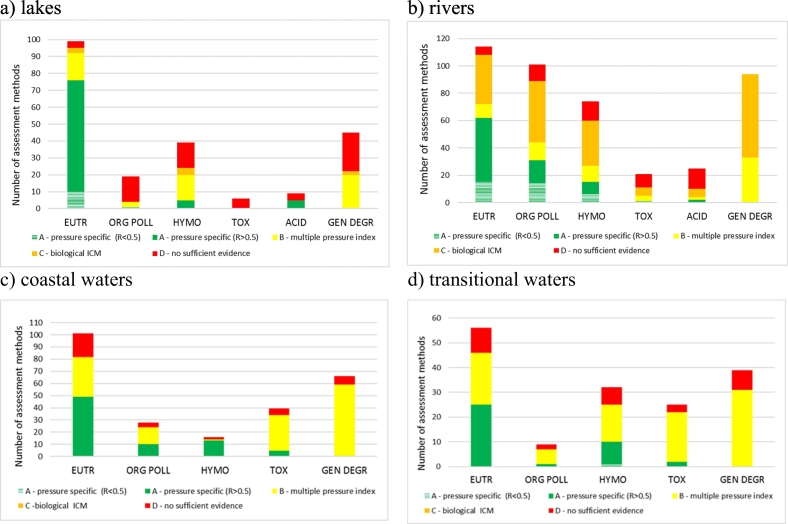
Number of national assessment methods for lakes, rivers, coastal and transitional waters addressing different pressures, with pressure-specific / multi-pressure / no relationships demonstrated. EUTR: eutrophication; HYMO: hydromorphology; ORG POLL: organic pollution; TOX: toxicity; ACID: acidification; GEN DEGR: general degradation. ICM: Intercalibration common metric.

The situation varies considerably between the different water categories. Fig. 3 shows the number of assessment methods addressing the main pressures for the different water categories, shown as the number of methods with pressure-specific/multi-pressure/no relationships demonstrated. For lakes, most methods address eutrophication (*n* = 99), with significantly fewer methods addressing hydromorphological alteration (*n* = 39) and “general degradation” (*n* = 46). Response to eutrophication is demonstrated for 96% of methods, including 77% with pressure-specific relationships; however, for several benthic invertebrates and fish methods and a few phytoplankton methods only multi-pressure relationships using combined indices were demonstrated. Response to hydromorphological alterations is demonstrated for 62% methods, including 49% with multi-pressure relationships but only five methods (13%) with pressure-specific relationships. Response to acidification is demonstrated in 55% methods, always with pressure-specific relationships.

For rivers, the three main pressures addressed are eutrophication (*n* = 114 methods), organic pollution (*n* = 101) and “general degradation” (*n* = 94), with fewer methods targeting hydromorphology (*n* = 74) and acidification (*n* = 25). Pressure–specific relationships are demonstrated for eutrophication (*n* = 62), while most other methods are demonstrated with multi-pressure indices.

For coastal waters, most of the methods address eutrophication (n = 101), “general degradation” (*n* = 59) and toxic pollution (n = 44); pressures less often addressed are organic pollution (*n* = 28), hydromorphology (*n* = 15) and invasive species (n = 4). Relationships with pressures are demonstrated for around 85% of the methods, with eutrophication (49%, mostly phytoplankton methods) and hydromorphology (12%, mostly angiosperms) being the pressures with the most pressure-specific relationships demonstrated.

In the case of transitional waters, all pressures are mostly addressed by all methods, generally via multi-pressure indices. Eutrophication (49%) and hydromorphology (35%) are the pressures most often demonstrated with pressure-specific relationships.

In conclusion, eutrophication is well covered and well demonstrated, while other pressures are less well or not covered and demonstrated mainly with multi-pressure relationships (except acidification where all methods are demonstrated with pressure-specific relationships).

### 3.3 Strength of pressure-response relationships

**Fig. 4:**
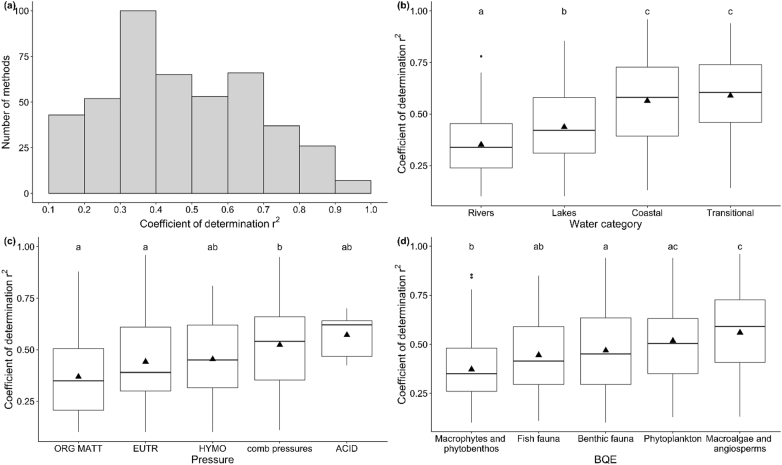
Range of determination coefficients (*r*^*2*^) from pressure-response relationships (a) all water categories, pressures, BQEs combined; (b) per water category; (c) per pressure assessed, (c) per BQE. Different letters indicate groups that are statistically different (*p* ≤ .05).

The explained variance (*r*^*2*^) spanned a wide range (<0.10–0.96), which is not surprising given the range in pressures, BQEs and datasets. The mean coefficient of determination was 0.45, and the median was 0.47. More than a half of *r*^*2*^ wewere in the range 0.20–0.50 (Fig. 4a). STtrength of pressure-response relationships differed significantly between water categories (Fig. 4b), pressures (Fig. 4c) and BQEs (Fig. 4d). Stronger relationships were reported for coastal and transitional waters (median *r*^*2*^ = 0.58 and *r*^*2*^ = 0.60, respectively) than for lakes (median *r*^*2*^ = 0.42) and rivers (median *r*^*2*^ = 0.33). The strongest relationships were recorded with acidification (median *r*^*2*^ = 0.62) however the number of methods is low (n = 6). The relationships with multi-pressure indices (median *r*^*2*^ = 0.54) were stronger than with the single pressures of eutrophication and organic pollution (median *r*^*2*^ = 0.39 and 0.35, respectively). The strongest pressure-response relationships were demonstrated using macroalgae and angiosperms (r^2^ = 0.59) and phytoplankton (*r*^*2*^ = 0.50), and the weakest pressure-response relationships were demonstrated using macrophytes and phytobenthos (*r*^*2*^ = 0.35).

### 3.4 BQEs used for different pressures

**Fig. 5:**
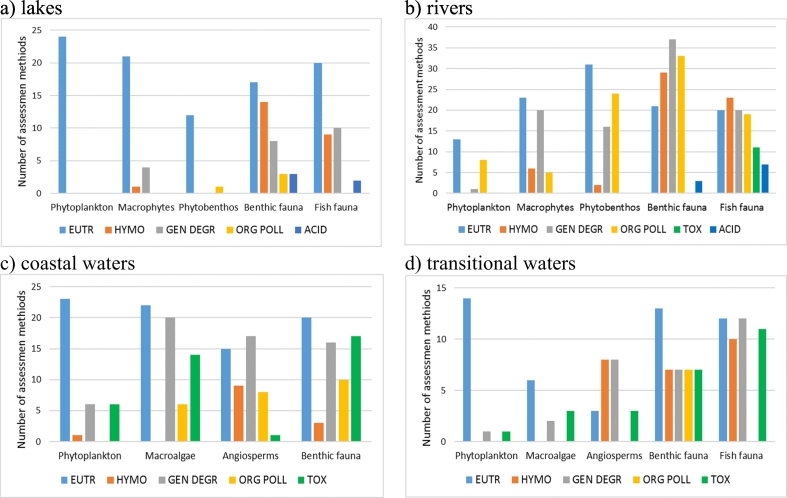
Sensitivity of BQE methods for different pressures for lakes, rivers, coastal and transitional waters (as reported by member states). EUTR: eutrophication; HYMO: hydromorphology; ORG POLL: organic pollution; TOX: toxicity; ACID: acidification; GEN DEGR: general degradation.

In lakes, primary producers are used mostly to detect eutrophication pressure, which is also assessed by benthic invertebrates and fish (Fig. 5). In contrast, other pressures (such as hydromorphology and acidification) are assessed only by benthic invertebrates and fish. Similarly, for rivers, primary producers are used for assessing eutrophication, with macrophytes also used to detect “general degradation” and phytobenthos to detect organic pollution (Fig. 5).

In coastal waters, most BQEs show relationships with eutrophication and general degradation. Hydromorphological pressure is addressed mainly by angiosperms, and toxic pollution by benthic invertebrates and macroalgae (Fig. 5).

In transitional waters, eutrophication is also addressed by all BQEs but mainly with phytoplankton, benthic invertebrates and fish. Surprisingly, there are fewer pressure-response relationships for macroalgae assessment methods compared to benthic invertebrate and fish methods. Hydromorphological pressure is addressed not only by angiosperms, but also by fish and benthic invertebrates (Fig. 5).

### 3.5 Pressures addressed by country

**Fig. 6:**
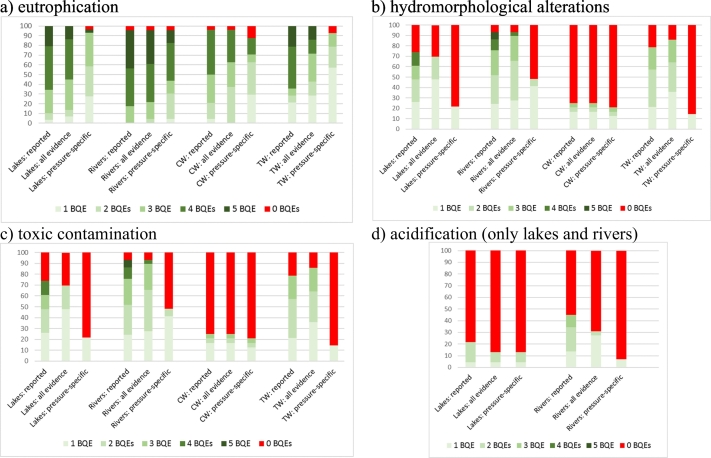
Number of Biological Quality Elements (BQEs) in national classification systems for lakes rivers, coastal (CW) and transitional waters (TW) sensitive for different pressures: (1) as reported by member states; (2) supported by some evidence (Table 1: Category A + B + C); (3) supported by pressure-specific evidence (Table 1: Category A). Y-axis shows the percentage of the total number of countries with relevant water bodies.

The range of pressures addressed differs between countries, as some, but not all countries have developed methods addressing / diagnosing all major pressures. For rivers and lakes, all countries have methods to detect eutrophication (most of them for four to five BQEs), which hold demonstrated pressure-specific relationships (most for three to five BQEs) (Fig. 6). In contrast, few countries have the capability to unequivocally detect hydromorphological alterations. Most countries report that their assessment methods can detect hydromorphological alterations in lakes and rivers but, in most cases, relationships are demonstrated via multi-pressure indices. Half of all countries for rivers and 80% of countries for lakes have not demonstrated pressure-specific relationships for hydromorphological alterations (Fig. 6).

Toxic pollution is addressed by half of all countries for rivers but only by four countries for lakes. However, pressure-specific relationships for toxic pollution have not been developed. For acidification, only two (rivers) and three (lakes) countries demonstrated pressure-specific relationships (Fig. 6).

All countries have methods for eutrophication in coastal and transitional waters (most addressed by three to four BQEs) and have demonstrated pressure-specific relationships in most cases (mostly for two BQEs in coastal and one in transitional waters) (Fig. 6). "General degradation" and toxic pollution are also addressed by most country's methods, with hydromorphology being the pressure least often addressed in coastal waters, and organic pollution being the pressure least often addressed in transitional waters. Only a few countries have demonstrated pressure-response relationships for hydromorphological alterations and toxic pollution.

## 4 Discussion

### 4.1 Pressures addressed by the assessment methods

#### 4.1.1 Eutrophication and organic pollution

Eutrophication is the pressure best covered for all four water categories (Fig. 2, Fig. 3). For >70% of the countries these pressures are addressed by three BQEs or more (Kelly et al., 2016). Pressure-specific evidence for eutrophication is well demonstrated for all water categories (Fig. 3).

As expected, phytoplankton and aquatic flora are the BQEs most sensitive to eutrophication (Lyche Solheim et al., 2013; Zaldívar et al., 2008). For these BQEs, pressure-response is demonstrated with direct nutrient-BQE relationships (Carvalho et al., 2013; Kelly et al., 2014; Poikane et al., 2018) underpinned by a strong conceptual framework and empirical research. However, benthic invertebrates and fish methods have also been developed specifically to tackle eutrophication (benthic invertebrates: Ruse, 2010; fish fauna: Argillier et al., 2013). In contrast to phytoplankton and aquatic flora, most of the benthic invertebrates and fish methods have shown their sensitivity to the pressures through a multi-pressure index rather than direct nutrient-BQE relationships (Lepage et al., 2016; Poikane et al., 2017).

Organic pollution is clearly addressed by benthic invertebrates in rivers, coastal and transitional waters (Borja et al., 2011, Borja et al., 2015). Most benthic invertebrate assessment methods are based on the percentage of opportunistic and sensitive species, and the proportion of the different species groups is highly correlated with the organic enrichment gradient (Borja et al., 2000; Pearson and Rosenberg, 1978; Warwick and Clarke, 1994). In practice, delimitation of eutrophication and organic pollution is not straightforward, as factors such as oxygen depletion may arise both directly from decay of autochthonous primary production or indirectly via heterotrophic breakdown of allochthonous inputs.

It is reasonable to expect, nonetheless, that effects of eutrophication and organic pollution are picked up by the biological assessment methods, and that biological indicators should guide how management targets are set (Poikane et al., 2019; Salas Herrero et al., 2019). There is, however, considerable uncertainty, especially for rivers and in multi-pressure situations (Phillips et al., 2019).

### 4.1.2 Hydromorphological pressures

In Europe, hydromorphological alterations are among the most significant pressures, affecting around 40% of surface water bodies, with the highest proportion reported for rivers and transitional waters (EEA [European Environment Agency], 2018). Hydromorphological pressures are addressed by at least one BQE in 90% of the countries for rivers, 75% of the countries for lakes and transitional waters, and in only 25% of the countries for coastal waters. Fish and benthic invertebrates are the BQEs responding most strongly for rivers and lakes (Lorenz et al., 2004; Urbanič, 2014), while angiosperms and benthic invertebrates are used in coastal and transitional waters (Orlando-Bonaca et al., 2012; Recio et al., 2013), and fish in transitional waters (Lepage et al., 2016).

For rivers and lakes, there is less evidence of a response to hydromorphological pressures than for eutrophication, as pressure-specific relationships (at least one BQE) have been developed by only 20% of the countries for lakes and 50% for rivers. Pressure-response relationships are often based on proxy indicators such as land use or multi-pressure indices (Poikane et al., 2016, Poikane et al., 2017; Segurado et al., 2014). Methods thus rarely respond to hydromorphological pressures alone, although there are a few exceptions (some countries have specific modules in their river benthic invertebrates methods designed to detect hydromorphological alterations: Böhmer et al., 2004) and a few countries have developed hydromorphology-specific methods for lakes (Poikane et al., 2016; Urbanič, 2014).

Similarly, only 15–20% of countries have developed pressure-specific relationships for coastal and transitional waters. Seagrasses and saltmarsh assessment methods for most North-East Atlantic countries show a relationship with hydromorphological pressures. In fact, hydromorphological pressures such as shoreline reinforcement or percentage of dredged area were used as an ICM for seagrass and saltmarsh intercalibration (Neto et al., 2018, Neto et al., 2019). In the case of fish, hydromorphological pressure is one of the components of indices used to test the response of assessment methods to pressures (Lepage et al., 2016). Intertidal area lost, realignment schemes, land claim, gross change in bathymetry and topography and interference with the hydrographical regime are some of the hydromorphological pressure indicators included in the common pressures index used for intercalibration for North-East Atlantic seagrasses and saltmarshes (Lepage et al., 2016).

In summary, there is not much evidence that the BQE methods currently in use reliably pick up the effects, as assessment methods have limited diagnostic capability: The situation varies between water bodies:

• Several countries lack methods (not even one for a single BQE) for assessing hydromorphological pressures: coastal waters (75% of countries), transitional waters (21%), lakes (26%), rivers (7%);
• Pressure-specific relationships are available only in a few countries, such relationships are missing in coastal waters (for 79% of countries), transitional waters (86%), lakes: (88%) and rivers (52%).

It is therefore important to use hydromorphological assessment methods alongside the BQEs, as well as develop appropriate specific hydromorphological ecological assessment methods.

### 4.1.3 Toxic pollution

Few assessment methods are reported to be sensitive to toxic pollution (either priority substances or river basin specific substances) for rivers, lakes and coastal waters. For lakes, no pressure-specific relationships have been developed, however, assessment methods for fish in rivers and transitional waters show relationships to pressure indices, which include toxic pollution (Lepage et al., 2016; Segurado et al., 2014).

In coastal waters, specific relationships have been demonstrated between benthic invertebrate methods and heavy metals concentrations in sediments only in five countries. Other methods have shown only indirect evidence (i.e. relationship with multi-pressure index) of their sensitivity to toxic substances (Borja et al., 2015). The absence of specific relationships is likely to be due to the scarce data on toxicants. For example, countries involved in the North-East Atlantic region intercalibration exercise showed direct relationships between their benthic invertebrate methods and copper, albeit using data provided by just one country (UK) (Muxika et al., 2019; van Hoey et al., 2019). However, in a recent meta-analysis with some of the most used transitional and coastal macroinvertebrate indices (AMBI and M-AMBI), correlations with several metals and toxic organic compounds, as well as different pressures, were demonstrated (Borja et al., 2019).

For transitional waters the situation is different: here there is ample evidence that the AMBI-based benthic invertebrate methods used by many countries respond to contamination by heavy metals (Borja et al., 2019). However, only France has shown pressure-specific relationships between fish assessment methods and heavy metals concentrations in mussels (Delpech et al., 2010), whilst Romania has shown a relationship between their benthic invertebrate method and annual mean water column concentrations for total heavy metals, total petroleum hydrocarbons, polyaromatic hydrocarbons and pesticides (Todorova et al., 2018).

We therefore conclude that current assessment methods do not reliably detect effects of toxic pollution:

• Significant number of countries lack methods (not even for one BQE) for assessing toxic pollution: coastal and transitional waters: 21%, lakes: 75%, rivers: 52%.;
• Pressure-specific relationships are available only in a few countries, such relationships are missing in coastal waters: 79% countries; transitional waters: 85%; lakes: 100%, and rivers: 97%.

Targeted methods (e.g. effect-based tools) could be developed to better integrate the effects of different toxicants in biological assessments.

### 4.1.4 Acidification

Acidification is a problem most often reported from rivers and lakes in Northern Europe, especially Scandinavian countries, UK and Ireland (Aherne and Curtis, 2003; Posch et al., 2019). However, a recent study has shown that acidified surface waters may occur particularly in the following regions (Austnes et al., 2018): the Pyrenees, border regions of Belgium, Luxembourg, France and Germany, mountainous regions on the borders of the Czech Republic (see Oulehle et al., 2017), Germany and Austria (see Vrba et al., 2016), the Tatra Mountains, the Italian Alps and northern Croatia.

Only three countries have fish and benthic invertebrate methods targeted to detect the effects of acidification, with strong evidence for pressure-impact relationships provided for two countries for rivers and three for lakes. It can therefore be concluded that acidification is well covered for some countries (Sweden, Norway and UK) whilst other countries with acidification-prone regions (especially Finland and Ireland) currently do not have any method focused on this pressure.

### 4.1.5 “General degradation” and “land use”

“General degradation” and “land use” are frequently mentioned as pressures addressed by BQEs, especially for rivers, coastal and transitional waters where it is often difficult to establish clear pressure-impact relationships. There is no common definition of “general degradation”, typically single pressures such as nutrients, organic matter, various hydromorphological alterations, and others are combined into a single pressure index (Borja et al., 2015). However, the list of pressures included and parameters used vary between regions and BQEs. For instance, a pressure index for fish-based methods in lakes of Central Europe includes human lake use (fishing, boating, bathing; Poikane et al., 2017) whilst a pressure index for fish-based methods in Mediterranean rivers includes acidification and toxic pollution (Segurado et al., 2014). Most of the Baltic countries use the Baltic Sea Pressure Index, an index developed for the evaluation of the distribution of pressures in that sea, including a total of seven types of pressure (Korpinen et al., 2012). In the case of the North-East Atlantic countries, different pressure indices are used, depending of the BQE, but always including eutrophication parameters, toxicants and hydromorphological pressures (Borja et al., 2011; Lepage et al., 2016). In the Mediterranean and Black Seas, most of the assessment methods show relationships with land use, specifically with the Land Uses Simplified Index (LUSI; Flo et al., 2019). This index is used as a measure of the total pressure (urban land use, agriculture, rivers, industries) to assess the relationship between assessment methods (usually, phytoplankton, macroalgae and macroinvertebrates) and environmental pressures (Camp et al., 2015; Pinedo et al., 2015; Torras et al., 2016). The problem with this kind of index is that it is not always clear which pressure is responsible for an observed response and, consequently, in cases of failure to achieve good status, it is not clear which pressures should be addressed (Carvalho et al., 2019; Lemm et al., 2019), although in some cases the analyses are successful in disentangling the effects of different pressures in the ecological status in transitional waters fish (Teichert et al., 2016). On the other hand, such approaches are reasonably successful at detecting a departure from good ecological status and, thus, at minimizing the risk of a “false negative” due to the absence of an appropriate pressure-specific metric.

“Land use” can be seen as a proxy for multiple pressures, including pollution, hydromorphological alterations, and other pressures, such as recreation. Many methods have relationships with land-use, as Corine Land Cover data have been readily available (in contrast to hydromorphological condition and toxic substances), and this approach was recommended by the Intercalibration Guidance (EC, 2011). However, the problem here is the same as with pressure indices: in cases of less than good status the cause of degradation and management measures are not obvious (it is clear that with 100% natural land cover water bodies will be in high or good status, and with high urban cover water bodies will be in less than good status – but what to do next?).

Benthic invertebrate and fish multimetric assessment methods typically show good relationships with such general pressure indices (Borja et al., 2011; Lepage et al., 2016). In some cases, a multimetric index is a combination of different modules or metrics specifically designed to detect effects of different pressures (Borja et al., 2015). In this case, the effects of different pressures can be disentangled using these different modules (Baattrup-Pedersen et al., 2019; Böhmer et al., 2004). In all other cases, relationships only provide general answers to pressure combinations, and hydromorphological and physicochemical supporting elements have to be used to detect the cause and suggest restoration measures (see Teichert et al., 2016).

### 4.2 Strengths and limitations of this study

The strength of this study is the detailed collation of data on biological assessment methods and demonstrations of pressure-response relationships for all countries, water categories and BQEs in the EU, along with Norway, who also implement EU environmental legislation. In the analysis, the differentiation has been made between single- and multi-pressure relationships demonstrated by biological assessment methods. This allowed (1) identification of pressures which might remain undetected by biological assessment; (2) evaluation of capacity of assessment methods to diagnose the cause of degradation.

One limitation for the study is that the database, includes only methods which are actually used in the ecological assessment which form part of a formal classification and reporting process. However, many other methods have been published and many of them could fill in gaps identified in this study, e.g. acidification in rivers (Juggins et al., 2016), hydromorphological pressures in lakes (Miler et al., 2013; Mjelde and Hellsten, 2013), toxic pollution in rivers (Archaimbault et al., 2010). We also suspect (and in some cases know) that such “off-piste” approaches are used to inform local decision-making.

A key question that arises, then, is whether the pattern of ecological status arising from the EU's formal reporting processes would change if the gaps identified in this paper were filled. Kelly et al. (2016) demonstrated a decreasing “marginal utility” when several BQEs were used to assess the same pressure (eutrophication in lakes). There were, nonetheless, impacted lakes detected using two BQEs that were missed when only one was used, so it should not be assumed that countries with only one assessment method tuned to a particular pressure will not have a number of “false negatives” among their assessment results. The scale of this effect will vary between countries and pressures, but should not be dismissed without further investigation.

## 5 Conclusions

In this study, we argue that the various pressures affecting European surface waters can be best managed if addressed properly by biological assessment methods. In particular, we argue that all major pressures must be addressed by biological assessment, and that a diagnostic capability should be available for such pressures. With no appropriate methods in use, some pressures may go undetected, thus not triggering restorative actions. Without diagnostic tools for specific pressure, there can be no informed choice of the management measure(s) needed to achieve good ecological status.

Our main findings can be summarized as follows:

• A huge number of assessment methods has been developed and intercalibrated; however, not all pressures have been addressed and indicators cannot, in most cases, diagnose the cause(s) of ecological degradation;
• Eutrophication is the pressure best covered by the assessment methods for all four water categories; pressure-impact relationships are reported for most BQEs for all water categories;
• In contrast, there is not much evidence that the assessment methods reliably pick up the effects of hydromorphological alterations, as only a few methods have clearly documented responses to hydromorphological degradation;
• Similarly, in the majority of cases, current assessment methods are not capable of reliably diagnosing ecological degradation caused by toxic pollution.

Therefore, we recommend that countries re-examine: (1) those pressures which affect different water categories in the country; (2) relevant assessment methods to tackle those pressures; (3) whether pressure-response relationships have been developed for each of these. Such an overview will indicate the problems with current biological assessment methods and guide future research directions.

## CRediT authorship contribution statement

**Sandra Poikane:** Conceptualization, Methodology, Formal analysis, Investigation, Data curation, Writing - original draft, Writing - review & editing, Visualization. **Fuensanta Salas Herrero:** Conceptualization, Methodology, Formal analysis, Investigation, Data curation, Writing - original draft, Writing - review & editing, Visualization. **Martyn G. Kelly:** Conceptualization, Writing - review & editing. **Angel Borja:** Conceptualization, Writing - review & editing. **Sebastian Birk:** Conceptualization, Writing - review & editing. **Wouter van de Bund:** Conceptualization, Methodology, Formal analysis, Investigation, Data curation, Writing - original draft, Writing - review & editing, Visualization.

## Declaration of competing interest

The authors declare that they have no known competing financial interests or personal relationships that could have appeared to influence the work reported in this paper.

## Appendix A Supplementary data

Overview of national biological assessment methods Image 1

## Footnotes

1 27 EU member states with the addition of Norway (which implements the WFD according to the European Economic Area agreement) and the United Kingdom (which left the EU during the course of this study)
