# Supplementary material for: European aquatic ecological assessment methods: A critical review of their sensitivity to key pressures
Source: Sci Total Environ. 2020 Oct 20;740:140075. doi: 10.1016/j.scitotenv.2020.140075 (PMC7456781; doi:10.1016/j.scitotenv.2020.140075)
Supplement: Supplementary file 1 — Overview of national biological assessment methods [file mmc1.docx]

# **European aquatic ecological assessment methods: a critical review of their sensitivity to key pressures**

# Sandra Poikane*^a^, Fuensanta Salas Herrero^a^, Martyn G. Kelly^b,c^, Angel Borja^d^, Sebastian Birk^e^, Wouter van de Bunda

# ^a^ European Commission Joint Research Centre (JRC), via Fermi 2749, Ispra, 21027 Italy

# ^b^ Bowburn Consultancy, 11 Monteigne Drive, Bowburn, Durham DH6 5QB, United Kingdom

# ^c^ School of Geography, Nottingham University, Nottingham NG7 2RD, United Kingdom

# ^d^ AZTI, Marine Research, Basque Research and Technology Alliance (BRTA), Herrera Kaia Portualdea s/n, 20100 Pasaia, Spain

# ^e^ Department of Aquatic Ecology, Faculty of Biology, University of Duisburg-Essen, 45141 Essen, Germany

# *Corresponding author; email: sandra.poikane@ec.europa.eu

# **Table S1. Overview of national lake assessment methods**

| **Biological quality element** | **Member state** | **Name of the method** | **Notes** |
| --- | --- | --- | --- |
| Benthic invertebrates | Austria | Multi-metric index for lakes in Austria (MILA) | Intercalibrated after EC Decision 2018 |
|  | Belgium-Flanders | Multimetric Macroinvertebrate Index Flanders (MMIF) |  |
|  | Bulgaria | HMMI_lakes (Hungarian Macrozoobenton Multimetric Index for Lakes) |  |
|  | Denmark | Danish Lake Macroinvertebrate Index (DLMI) | Intercalibrated after EC Decision 2018 |
|  | Estonia | Estonian surface water ecological quality assessment – Lake macroinvertebrates |  |
|  | Finland | Revised Finnish lake invertebrate fauna assessment method (PICM) | Intercalibrated after EC Decision 2018 |
|  | Germany | AESHNA - Bewertungsverfahren für das eulitorale Makrozoobenthos in Seen zur Umsetzung der EG-Wasserrahmenrichtlinie in Deutschland  (lowland types) | Method used in Central ecoregion |
|  | Germany | AESHNA - Bewertungsverfahren für das eulitorale Makrozoobenthos in Seen zur Umsetzung der EG-Wasserrahmenrichtlinie in Deutschland (alpine types) | Method used in Alpine ecoregion |
|  | Greece | Greek Lake Benthic invertebrate Index (GLBiI) |  |
|  | Hungary | HMMI_lakes (Hungarian Macrozoobenton Multimetric Index for Lakes |  |
|  | Italy | BQIES (Benthic Quality Index Expected Species number) |  |
|  | Latvia | Latvian Lake Macroinvertebrate Multimetric Index (LLMMI) |  |
|  | Lithuania | Lithuanian Lake Macroinvertebrate Index |  |
|  | Netherlands | WFDi - Metric for Natural Watertypes |  |
|  | Norway | Multimetric Invertebrate Index for Clear Lakes (MultiClear) |  |
|  | Poland | Lake Macroinvertebrate Index (LMI) | Intercalibrated after EC Decision 2018 |
|  | Romania | ECO-NL-BENT Romanian ecological status assessment system for natural lakes using benthic invertebrates |  |
|  | Slovenia | Metodologija vrednotenja ekološkega stanja jezer z bentoškimi nevretenčarji v Sloveniji |  |
|  | Spain | Spanish Invertebrates Index for Lakes (IBCAEL) | Intercalibrated after EC Decision 2018 |
|  | Spain | QAELS_2010_ index | Intercalibrated after EC Decision 2018 |
|  | Sweden | Benthic Quality Index (BQI) | Method for eutrophication |
|  | Sweden | Multimetric Invertebrate L)ake Acidification index (MILA) | Method for acidification |
|  | United Kingdom | Chironomid Pupal Exuvial Technique (CPET) | Method for eutrophication |
|  | United Kingdom | LAMM (Lake Acidification Macroinvertebrate Metric) | Method for acidification |
|  |  |  |  |
| Fish fauna | Austria | ALFI (Austrian lake fish index): A multimetric index to assess the ecological status of alpine lakes based on fish fauna |  |
|  | Belgium -Flanders | Fish-based index for lakes and reservoirs in Flanders (Belgium) |  |
|  | Bulgaria | Bulgarian fish based method for ecological classification and monitoring of lakes |  |
|  | Denmark | Danish Lake Fish Index |  |
|  | Estonia | LAFIEE index |  |
|  | Finland | EQR4 index |  |
|  | France | European Lake Fish Index (ELFI) |  |
|  | Germany | ALP DeLFI_SITE - Deutsches probennahmestandort-spezifisches Bewertungsverfahren für Fische in Seen zur Umsetzung der EG-Wasserrahmenrichtlinie | Method used in Central ecoregion |
|  | Germany | CB DeLFI_SITE - Deutsches probennahmestandort-spezifisches Bewertungsverfahren für Fische in Seen zur Umsetzung der EG-Wasserrahmenrichtlinie | Method used in Alpine ecoregion |
|  | Greece | Greek Lake Fish Index (GLFI) |  |
|  | Ireland | FIL2 index |  |
|  | Italy | Lake Fish Index (LFI) |  |
|  | Latvia | Latvian Lake Fish Index |  |
|  | Lithuania | Lithuanian Lake Fish Index |  |
|  | Netherlands | National fish assessment system of the Netherlands (VISMAATLAT) |  |
|  | Norway | AindexW5 | Method for acidification |
|  | Norway | EindexW3 | Method for eutrophication |
|  | Poland | National fish assessment system of Poland based on fisheries statistics (LFI+ index) |  |
|  | Poland | National fish assessment system of Poland based on gillnet sampling (LFI EN index) |  |
|  | Slovenia | Metodologija vrednotenja ekološkega stanja jezer na podlagi rib (Ecological status assessment system for lakes using fish in Slovenia) | intercalibrated after EC Decision 2018 |
|  | Sweden | AindexW5 | Method for acidification |
|  | Sweden | EindexW3 | Method for eutrophication |
|  | United Kingdom | FIL2 index |  |
|  |  |  |  |
| Macrophytes | Austria | AIM for Lakes (Austrian Index Macrophytes for lakes) |  |
|  | Belgium - Flanders | Flemish macrophyte assessment system |  |
|  | Bulgaria | RI-BG - Adopted Reference Index |  |
|  | Denmark | Danish Lake Macrophytes Index |  |
|  | Estonia | Estonian surface water ecological quality assessment – lake macrophytes |  |
|  | Finland | Finnish macrophyte classification system (Finnmac) |  |
|  | France | French Macrophyte Index for Lakes (IBML) Indice Biologique Macrophytique en Lacs |  |
|  | Germany | Verfahrensanleitung für die ökologische Bewertung von Seen zur Umsetzung der EG-Wasserrahmenrichtlinie: Makrophyten und Phytobenthos (PHYLIB), Modul Makrophyten |  |
|  | Greece | HeLM - Hellenic Lake Macrophytes Assessment Method |  |
|  | Hungary | HU-RI - Adopted Reference Index |  |
|  | Ireland | Free Macrophyte Index |  |
|  | Italy | MacroIMMI (Macrophytic index for the evaluation of the ecological quality of the Italian lakes) |  |
|  | Italy | VLMMI - Volcanic Lakes Multimetric Macrophyte Index |  |
|  | Latvia | Latvian macrophyte assessment method |  |
|  | Lithuania | Lithuanian Lake Macrophyte Index |  |
|  | Netherlands | WFD-metrics for natural water types |  |
|  | Norway | National macrophyte index (Trophic Index – TIc) |  |
|  | Poland | Macrophyte based indication method for lakes - Ecological Status Macrophyte Index ESMI |  |
|  | Romania | MIRO - Macrophyte Index for Romanian Lakes (Adapted Reference Index) |  |
|  | Slovenia | SMILE (Slovenian macrophyte-based index for lake ecosystems) |  |
|  | Spain | Spanish Macrophytes assessment method for Lakes (OFALAM) | Intercalibrated after EC Decision 2018 |
|  | Sweden | Trophic Macrophyte Index (TMI) |  |
|  | United Kingdom | Lake LEAFPACS 2 |  |
|  |  |  |  |
| Phytobenthos | Belgium - Flanders | Proportions of Impact-Sensitive and Impact-Associated Diatoms (PISIAD) |  |
|  | Finland | Finnish lake phytobenthos method |  |
|  | Germany | Verfahrensanleitung für die ökologische Bewertung von Seen zur Umsetzung der EG-Wasserrahmenrichtlinie: Makrophyten und Phytobenthos (PHYLIB), Modul Phytobenthos |  |
|  | Hungary | MIL- Multimetric Index for Lakes |  |
|  | Ireland | Lake Trophic Diatom Index (IE) |  |
|  | Italy | Italian national method for the evaluation of the ecological quality of lake waterbodies using benthic diatoms (EPI-L) |  |
|  | Lithuania | Lithuanian Lake Phytobenthos Index |  |
|  | Poland | PL IOJ (Multimetryczny Indeks Okrzemkowy dla Jezior = Multimetric Diatom Index for Lakes) |  |
|  | Romania | Romanian Diatom Index RDI for lowland lakes | Intercalibrated after EC Decision 2018 |
|  | Romania | National (Romanian) Assessment Method for Natural Lakes Ecological Status based on Phytobenthos (Diatoms) RO-AMLP |  |
|  | Slovenia | Trophic Index (TI) |  |
|  | Sweden | IPS index |  |
|  | United Kingdom | DARLEQ 2 |  |
|  |  |  |  |
| Phytoplankton | Austria | Evaluation of the biological quality elements, Part B2 – phytoplankton |  |
|  | Belgium - Flanders | Flemish phytoplankton assessment method for lakes |  |
|  | Bulgaria | HLPI-Hungarian lake phytoplankton index |  |
|  | Denmark | Danish Lake Phytoplankton Index |  |
|  | Estonia | Estonian surface water ecological quality assessment – lake phytoplankton |  |
|  | Finland | Finnish phytoplankton assessment method for lakes |  |
|  | France | Phytoplankton Index for Lakes (IPLAC) |  |
|  | Germany | PSI (Phyto-Seen-Index) - Bewertungsverfahren für Seen mittels Phytoplankton zur Umsetzung der EG-Wasserrahmenrichtlinie in Deutschland |  |
|  | Greece | HeLPhy - Hellenic Lake Phytoplankton Assessment Method |  |
|  | Greece | New Mediterranean Assessment System for Reservoirs (NMASRP) |  |
|  | Hungary | HLPI-Hungarian lake phytoplankton index |  |
|  | Ireland | IE Lake Phytoplankton Index |  |
|  | Italy | New Italian Method (NITMET) |  |
|  | Italy | Italian Phytoplankton Assessment Method (IPAM) |  |
|  | Latvia | Latvian Lake Phytoplankton Index |  |
|  | Lithuania | German Phytoplankton Index (PSI) |  |
|  | Netherlands | WFD - metrics for natural watertypes |  |
|  | Norway | Lake phytoplankton ecological status classification method |  |
|  | Poland | Phytoplankton method for Polish Lakes (PMPL) |  |
|  | Romania | HLPI-Hungarian lake phytoplankton index |  |
|  | Slovenia | Metodologija vrednotenja ekološkega stanja jezer s fitoplanktonom v Sloveniji (Ecological status assessment system for lakes using phytoplankton in Slovenia) |  |
|  | Spain | Mediterranean Assessment System for Reservoirs Phytoplankton (MASRP) |  |
|  | Sweden | Ecological assessment methods for lakes, Quality factor - phytoplankton |  |
|  | United Kingdom | Phytoplankton Lake Assessment Tool with Uncertainty Module (PLUTO) |  |

Table S2. Overview of national river assessment methods.

| **BQE** | **Member State** | **Name of the method** | **Notes** |
| --- | --- | --- | --- |
| **Benthic invertebrates** | Austria | Slovak assessment of benthic invertebrates in large rivers (for large lowland rivers) |  |
|  | Austria | Assessment of the biological quality elements - part benthic invertebrates |  |
|  | Belgium-Flanders | Multimetric Macroinvertebrate Index Flanders (MMIF) |  |
|  | Belgium-Wallonia | Indice Biologique Global Normalisé (IBGN) |  |
|  | Bulgaria | IBI (BG) (Irish Biotic Index (BG)) |  |
|  | Bulgaria | mRBA - Modified Rapid Biological Assessment (for very large rivers( |  |
|  | Croatia | Croatian assessment system based on benthic invertebrates in very large rivers |  |
|  | Cyprus | STAR Intercalibration Common Metric Index (STAR_ICMi) |  |
|  | Czech Republic | Czech system for ecological status assessment of rivers using benthic macroinvertebrates |  |
|  | Czech Republic | Czech system for ecological status assessment of large nonwadeable rivers using benthic macroinvertebrates |  |
|  | Denmark | Danish Stream Fauna Index (DSFI) |  |
|  | Estonia | Estonian surface water ecological quality assessment – river macroinvertebrates |  |
|  | Finland | Revised Finnish river invertebrate fauna assessment method | Intercalibrated after EC Decision 2018 |
|  | France | I2M2 index | Intercalibrated after EC Decision 2018 |
|  | Germany | PERLODES -Bewertungsverfahren von Fließgewässern auf Basis des Makrozoobenthos |  |
|  | Germany | PTI: Potamon-Typie-Index (for very large rivers) |  |
|  | Greece | Hellenic Evaluation System-2 (HESY-2) |  |
|  | Greece | STAR_ICMi index (for very large rivers) | Intercalibrated after EC Decision 2018 |
|  | Hungary | Hungarian Multimetric Macroinvertebrate Index |  |
|  | Ireland | Quality Rating System (Q-value) |  |
|  | Italy | MacrOper, based on STAR Intercalibration Common Metric Index (STAR_ICMi) |  |
|  | Latvia | Latvian Macroinvertebrate Index (LMI) |  |
|  | Latvia | Latvian large river macroinvertebrate index LRMI |  |
|  | Lithuania | Lithuanian River Macroinvertebrate Index (LRMI) |  |
|  | Luxembourg | Classification luxembourgeoise DCE Indice Biologique Global Normalisé (IBG) |  |
|  | Netherlands | KRW-maatlat |  |
|  | Norway | Norway ASPT – Average Score per Taxobn | For general degradation |
|  | Norway | AcidIndex2 (Modified Raddum index2) ( | For acidification |
|  | Poland | RIVECOmacro - MMI_PL |  |
|  | Portugal | Rivers Biological Quality Assessment Method-Benthic Invertebrates (IPtIN, IPtIS) |  |
|  | Romania | Assessment method for ecological status of water bodies based on macroinvertebrates |  |
|  | Romania | ECO-BENT index (for very large rivers) |  |
|  | Slovakia | Slovak assessment of benthic invertebrates in rivers |  |
|  | Slovakia | Slovak assessment of benthic invertebrates in large rivers |  |
|  | Slovenia | Metodologija vrednotenja ekološkega stanja rek z bentoškimi nevretenčarji v Sloveniji |  |
|  | Spain | Iberian Mediterranean Multimetric Index using quantitative data (IMMi-T) | Mediterranean  ecoregion |
|  | Spain | Iberian Biological Monitoring Working Party (IBMWP) |  |
|  | Spain | METI index | Central Baltic ecoregion |
|  | Spain | INVMIB index (INVertebrate Multimetric Illes Balears) | Balearic islands, intercalibrated after EC Decision 2018 |
|  | Sweden | ASPT index | For general degradation |
|  | Sweden | DJ-index | For general degradation |
|  | Sweden | MISA: Multimetric Invertebrate Stream Acidification index | For acidification |
|  | United Kingdom | River Invertebrate Classification Tool (RICT)- WHPT | For general degradation |
|  | United Kingdom | WFD-AWIC index | For acidification |
| **Fish fauna** | Austria | Austrian Fish index (FIA) |  |
|  | Belgium-Flanders | Upstream and Lowland IBI index |  |
|  | Belgium-Wallonia | IBIP index |  |
|  | Bulgaria | TsBRI (Type Specific Bulgarian Fish Index ) |  |
|  | Czech Republic | Czech multimetric method (CZI) |  |
|  | Finland | Finnish Fish Index (FiFi) |  |
|  | France | FBI (Fish-Based index): Classification française DCE Indice Poissons Rivière (IPR) |  |
|  | Germany | FIBS – fischbasiertes Bewertungssystem für Fließgewässer zur Umsetzung der EG-Wasserrahmenrichtlinie in Deutschland |  |
|  | Greece | Hellenic Fish Index (HeFI) |  |
|  | Hungary | Hungarian Multimetric Fish Indices (HMMFI) | Intercalibrated after EC Decision 2018 |
|  | Ireland | Fish Classification Scheme 2 Ireland (FCS2) |  |
|  | Italy | NISECI index (New Index of Ecological Status of Fish Communities) |  |
|  | Latvia | Latvian Fish Index |  |
|  | Lithuania | Lithuanian River Fish Index |  |
|  | Luxembourg | Classification française DCE Indice Poissons Rivière (IPR) |  |
|  | Netherlands | NLFISR index |  |
|  | Poland | EFI+PL index |  |
|  | Portugal | F-IBIP - Fish-based Index of Biotic Integrity for Portuguese Wadeable Streams |  |
|  | Romania | EFI+ European Fish index |  |
|  | Slovakia | Fish Index of Slovakia FIS |  |
|  | Slovenia | Metodologija vrednotenja ekološkega stanja vodotokov na podlagi rib (SIFAIR index) |  |
|  | Spain | IBIMED index |  |
|  | Sweden | Swedish method VIX |  |
|  | United Kingdom | FCS2 index |  |
|  |  |  |  |
| **Macrophytes** | Austria | AIM for Rivers (Austrian Index Macrophytes for rivers) |  |
|  | Belgium-Flanders | MAFWAT - Flemish macrophyte assessment system |  |
|  | Belgium-Wallonia | IBMR-WL - Biological Macrophyte Index for Rivers |  |
|  | Bulgaria | Reference Index |  |
|  | Cyprus | IBMR – Biological Macrophyte Index for Rivers |  |
|  | Czech Republic | Assessment method of surface running water bodies in the Czech Republic using biological quality element macrophytes |  |
|  | Denmark | DSPI - Danish Stream Plant Index |  |
|  | Estonia | Estonian assessment system for rivers using  Macrophytes (MIR) | Intercalibrated after EC Decision 2018 |
|  | Finland | Trophic index TIc |  |
|  | France | IBMR - Indice biologique macrophytique en rivière French standard NF T90-395 (2003-10-01) |  |
|  | Germany | Verfahrensanleitung für die ökologische Bewertung von Fließgewässern zur Umsetzung der EG-Wasserrahmenrichtlinie: Makrophyten und Phytobenthos (PHYLIB), Modul Makrophyten |  |
|  | Germany | NRW-Verfahren zur Bewertung von Fließgewässern mit Makrophyten |  |
|  | Greece | IBMR – Biological Macrophyte Index for Rivers |  |
|  | Hungary | Reference Index |  |
|  | Ireland | MTR - IE - Mean Trophic Ranking |  |
|  | Italy | IBMR - IT - Biological Macrophyte Index for Rivers |  |
|  | Latvia | Latvian assessment method using macrophytes |  |
|  | Lithuania | Lithuanian River Macrophyte Index |  |
|  | Luxembourg | IBMR - LU - Biological Macrophyte Index for Rivers |  |
|  | Netherlands | Revised assessment method for rivers in The Netherlands using macrophytes |  |
|  | Norway | Trophic index TIc |  |
|  | Poland | MIR - Macrophyte Index for Rivers |  |
|  | Portugal | IBMR - Biological Macrophyte Index for Rivers |  |
|  | Romania | Romanian Macrophyte-based assessment system for rivers (Macrophyte River Index (MARI)) |  |
|  | Slovakia | Macrophyte Biological Index for Rivers (IBMR-SK) |  |
|  | Slovenia | River Macrophyte Index |  |
|  | Spain | IBMR – Biological Macrophyte Index for Rivers |  |
|  | Sweden | Trophic index TIc |  |
|  | United Kingdom | River LEAFPACS 2 |  |
|  |  |  |  |
| **Phytobenthos** | Austria | Assessment of the biological quality elements - part phytobenthos |  |
|  | Belgium-Flanders | Proportions of Impact-Sensitive and Impact-Associated Diatoms (PISIAD) |  |
|  | Belgium-Wallonia | IPS index |  |
|  | Bulgaria | IPS index |  |
|  | Croatia | Ecological status assessment system for phytobenthos in rivers based on diatoms |  |
|  | Cyprus | IPS index |  |
|  | Czech Republic | Czech assessment method for rivers using phytobenthos |  |
|  | Estonia | Indice de Polluosensibilité Spécifique (IPS) |  |
|  | Estonia | Estonian phytobenthos assessment system for very large rivers |  |
|  | Finland | Finnish river phytobenthos method |  |
|  | France | IBD 2007 (Coste et al, Ecol. Ind. 2009). AFNOR NF-T-90-354, December 2007. Arrêté ministériel du 25 janvier 2010 modifié relatif aux méthodes et critères d’évaluation de l’état écologique {...} des eaux de surface |  |
|  | Germany | Verfahrensanleitung für die ökologische Bewertung von Fließgewässern zur Umsetzung der EG-Wasserrahmenrichtlinie: Makrophyten und Phytobenthos (PHYLIB), Modul Diatomeen |  |
|  | Greece | IPS (Coste in Cemagref, 1982) Intercalibrated (EQR IPS) |  |
|  | Hungary | Ecological status assessment for rivers based on diatoms | Intercalibrated after EC Decision 2018 |
|  | Ireland | Revised Trophic Diatom Index (TDI) |  |
|  | Italy | ICMi (Intercalibration Common Metric) Index |  |
|  | Lithuania | Lithuanian Phytobenthos Index |  |
|  | Luxembourg | Indice de Polluosensibilité Spécifique (IPS) |  |
|  | Netherlands | KRW Maatlat |  |
|  | Norway | Periphyton Index of Trophic Status (PIT) |  |
|  | Poland | Indeks Okrzemkowy IO dla rzek (Diatom Index for rivers) |  |
|  | Portugal | IPS (Coste in Cemagref, 1982) |  |
|  | Romania | National (Romanian) Assessment Method for Rivers Ecological Status based on Phytobenthos (Diatoms) RO-AMRP |  |
|  | Romania | Romanian Assessment Method for Very Large Rivers Ecological Status based on Phytobenthos | Intercalibrated after EC Decision 2018 |
|  | Slovakia | Ecological status assessment system for rivers using phytobenthos |  |
|  | Slovenia | Metodologija vrednotenja ekološkega stanja rek s fitobentosom in makrofiti v Sloveniji; fitobentos (Ecological status assessment system for rivers using phytobenthos and macrophytes in Slovenia; Phytobenthos) |  |
|  | Spain | IPS index | Mediterranean region |
|  | Spain | Diatom multimetric index (MDIAT) | Central region |
|  | Spain | DIATome Multimetric Illes Balears (DIATMIB) index | Balearic islands, intercalibrated after EC Decision 2018 |
|  | Sweden | Swedish assessment methods, Swedish EPA regulations (NFS 2008:1) based on Indice de Polluosensibilité Spécifique (IPS) |  |
|  | United Kingdom | Diatom Assessment for River Ecological Status (DARLEQ2) |  |
|  |  |  |  |
| **Phytoplankton** | Austria | German PhytoFluss-Index 4.0 |  |
|  | Belgium -Flanders | German PhytoFluss-Index 2.0 |  |
|  | Bulgaria | German PhytoFluss-Index 4.0 |  |
|  | Croatia | HRPI - Hungarian River Phytoplankton Index |  |
|  | Czech Republic | CZ - Assessment method for ecological status of rivers based on phytoplankton |  |
|  | Estonia | EST_PHYPLA_R - Assessment system for rivers using phytoplankton |  |
|  | Germany | German PhytoFluss-Index |  |
|  | Hungary | HRPI - Hungarian River Phytoplankton Index |  |
|  | Latvia | Latvian Large River Phytoplankton Index |  |
|  | Lithuania | German PhytoFluss-Index for lowland rivers of type 15.2 |  |
|  | Poland | IFPL metric - Method for large rivers assessment using phytoplankton |  |
|  | Romania | ECO-FITO - Assessment Method for Ecological Status of the Water Bodies based on Phytoplankton |  |
|  | Slovakia | Phytoplankton-SK - Slovak assessment of phytoplankton in large rivers |  |

Table S3. Overview of national transitional waters assessment methods. MED – Mediterranean Sea Region, NEA - North-East Atlantic Sea Region

| **BQE** | **Member State** | **Name of the method** | **Notes** |
| --- | --- | --- | --- |
| Phytoplankton | Belgium | Chlorophyll a |  |
|  | Croatia | Multimetric Phytoplankton Index (MPI) |  |
|  | France (MED) | Phytoplankton index for Mediterranean poly-euhaline lagoons (PhIL) |  |
|  | France (NEA) | Chlorophyll a |  |
|  | Germany (NEA) | Chlorophyll a |  |
|  | Greece | Multimetric Phytoplankton Index (MPI) |  |
|  | Italy | Multimetric Phytoplankton Index (MPI) |  |
|  | Ireland | Chlorophyll a |  |
|  | Lithuania | Chlorophyll a |  |
|  | Netherlands | Chlorophyll a |  |
|  | Poland | Chlorophyll a |  |
|  | Portugal | Chlorophyll a |  |
|  | Romania | Integrated Biological index (IBI) |  |
|  | Spain (MED) | FITOHMIB Phytoplankton index for transitional waters (TWIf) |  |
|  | Spain (NEA) | Chlorophyll a |  |
|  | United Kingdom | Chlorophyll a |  |
| Benthic invertebrates fauna | Belgium | Benthic Ecosystem Quality Index (BEQI) |  |
|  | Croatia | Marine Biotic index (AMBI) | Method intercalibrated after EC Decision 2018 |
|  | France (MED) | Multivariate AZTI Marine Biotic Index (MAMBI) |  |
|  | Germany (NEA) | Aestuar Type Verfahre ( AeTV) | oligohaline stretches of the transitional waters |
|  | Germany (NEA) | Multivariate AZTI Marine Biotic Index (MAMBI) | mesohaline and polyhaline stretches |
|  | Greece | Multivariate AZTI Marine Biotic Index (MAMBI) |  |
|  | Italy | Multivariate AZTI Marine Biotic Index (MAMBI) |  |
|  | Ireland | Infauna Quality Index (IQI) |  |
|  | Netherlands | Benthic Ecosystem Quality Index 2 (BEQI2) |  |
|  | Portugal | Benthic assessment tool (BAT) |  |
|  | Romania | Multivariate AZTI Marine Biotic Index Normalized (MAMBI-n) |  |
|  | Spain (MED) | BO2A | Southern Coast (estuaries) |
|  | Spain (MED) | INVHMIB | Balearic Islands (coastal lagoons) |
|  | Spain (MED) | Water quality of lentic shallow environments (QAELS) | Northeastern coast (coastal lagoons) |
|  | Spain (NEA) | Multivariate AZTI Marine Biotic Index (MAMBI) | Basque country |
|  | Spain (NEA) | Taxonomically Sufficient Benthic Multimetric (TasBEM) | Andalusia region |
|  | Spain (NEA) | Quality of Soft Bottoms (QSB) | Cantabria region |
|  | United Kingdom | Infaunal Quality Index (IQI) |  |
| Macroalgae and angiosperms | Belgium | Tidal Marsh Quality Index (TMQI) |  |
|  | Croatia | ZonoMI index | Method intercalibrated after EC Decision 2018 |
|  | Germany (NEA) | Assessment tool for intertidal seagrass in coastal and transitional waters (SG-DE) | Method using seagrasses |
|  | Germany (NEA) | Assessment of saltmarsh vegetation in coastal and transitional waters (EM) | Methods using saltmarshes |
|  | France (MED) | EXCLAME |  |
|  | France (NEA) | Macroalgal Bloom Assessment (CWOGA) | Methods using opportunistic macroalgae |
|  | France (NEA) | Seagrass beds quality in coastal and transitional water bodies (SBQ) | Methods using seagrasses |
|  | Greece | Ecological Evaluation Index (EEI-c) |  |
|  | Ireland | Opportunistic Green Macroalgal Abundance (OGA tool) | Method using opportunistic macroalgae |
|  | Ireland | Saltmarsh Angiosperm Assessment Tool for Ireland (SMAATIE) | Methods using saltmarshes |
|  | Italy | Macrophyte Quality Assessment Index (MQAI) |  |
|  | Netherlands | WFD-metrics for natural watertypes: tidal saltmarsh (TSM) | Method using saltmarshes |
|  | Netherlands | Monitoring beds of SG per waterbody using arial photographs, ground truth and specifyting surface & density per species (SG-NL) | Method using seagrasses |
|  | Portugal | Angiosperm Quality Assessment Index (AQuA) | Method using saltmarshes |
|  | Portugal | Seagrass quality index (SQI) | Method using seagrasses |
|  | Spain (NEA) | Angiosperm Quality Index | Method using saltmarshes |
|  | United Kingdom | Opportunistic Macroalgae Blooming Tool (OMBT) | Method using opportunistic macroalgae |
|  | United Kingdom | UK Saltmarsh Tool (SM) | Method using saltmarshes |
|  | United Kingdom | Intertidal Seagrass tool (SG-UK) | Methods using seagrasses |
| Fish | Belgium | Zeeschelde Estuarine Biotic Index (EBI) |  |
|  | Croatia | Modified Estuarine Fish index (M-EFI) |  |
|  | France (NEA) | Estuarine and Lagoon Fish Index (ELFI) |  |
|  | Germany (NEA) | Fischbasiertes Bewertungswerkzeug für Übergangsgewässer der norddeutschen Ästuare (FAT-TW) |  |
|  | Italy | Habitat Fish Bio-Indicator (HFBI) |  |
|  | Ireland | Estuarine Multi-metric Fish Index (EMFI) |  |
|  | Netherlands | Fish index for transitional waters (FAT-TW-WFD) |  |
|  | Portugal | Estuarine Fish Assessment Index (EFAI) |  |
|  | Spain (NEA) | AZTI’s Fish Index (AFI) |  |
|  | United Kingdom | Estuarine Multi-metric Fish Index (EMFI)  Transitional Fish Classification Index (TFCI) |  |

Table S4. Overview of national coastal waters assessment methods.

| **BQE** | **Member State** | **Name of the method** | **Notes** |
| --- | --- | --- | --- |
| Phytoplankton | Belgium | Chlorophyll a |  |
|  | Bulgaria | Integrated Biological index (IBI) |  |
|  | Croatia | Chlorophyll a |  |
|  | Cyprus | Chlorophyll a |  |
|  | France (MED) | Chlorophyll a |  |
|  | France (NEA) | Chlorophyll a |  |
|  | Germany (Baltic) | Phytoplankton method |  |
|  | Germany (NEA) | Chlorophyll a |  |
|  | Greece | Chlorophyll a |  |
|  | Italy | Chlorophyll a |  |
|  | Ireland | Chlorophyll a |  |
|  | Lithuania | Chlorophyll a |  |
|  | Netherlands | Chlorophyll a |  |
|  | Norway | Chlorophyll a |  |
|  | Poland | Polish Phytoplankton method |  |
|  | Portugal | Chlorophyll a |  |
|  | Romania | Integrated Biological index (IBI) |  |
|  | Slovenia | Chlorophyll a |  |
|  | Spain (MED) | Chlorophyll a |  |
|  | Spain (NEA) | Chlorophyll a |  |
|  | Sweden (Baltic) | Chlorophyll a |  |
|  | Sweden (NEA) | Chlorophyll a |  |
|  | United Kingdom | Chlorophyll a | For the IC common type NEA 1/26 |
|  | United Kingdom | Phytoplankton method | For the IC common type NEA 7 |
| Benthic invertebrates fauna | Belgium | Benthic Ecosystem Quality Index (BEQI) |  |
|  | Bulgaria | Multivariate AZTI Marine Biotic Index Normalized (MAMBI-n) |  |
|  | Cyprus | BENTIX index |  |
|  | Denmark (Baltic) | Danish Quality Index (DKI) |  |
|  | Denmark (NEA) | Danish Quality Index (DKI) |  |
|  | Finland | Finnish Brackish water Benthic Index (BBI) |  |
|  | France (MED) | Marine Biotic Index (AMBI) |  |
|  | France (NEA) | Multivariate AZTI Marine Biotic Index (MAMBI) |  |
|  | Germany (Baltic) | Marine Biotic Index Tool (MARBIT) |  |
|  | Germany (NEA) | Multivariate AZTI Marine Biotic Index (MAMBI) |  |
|  | Greece | BENTIX index |  |
|  | Italy | Multivariate AZTI Marine Biotic Index (MAMBI) |  |
|  | Ireland | Infauna Quality Index (IQI) |  |
|  | Latvia | Benthic quality index (BQI-LV) |  |
|  | Lithuania | Lithuanian Benthic quality index (BQI-LT) |  |
|  | Netherlands | Benthic Ecosystem Quality Index 2 (BEQI2) |  |
|  | Norway | Norwegian Quality Index (NQI) |  |
|  | Poland | Macrozoobenthos BQE assessment by multimetric index (B) |  |
|  | Portugal | Benthic assessment tool (BAT) |  |
|  | Romania | Multivariate AZTI Marine Biotic Index Normalized (MAMBI-n) |  |
|  | Slovenia | Multivariate AZTI Marine Biotic Index (MAMBI) |  |
|  | Spain (MED) | Mediterráneo Occidental index (MEDOCC)  Benthic Opportunistic polychaetes/amphipods index (BOPA) |  |
|  | Spain (NEA) | Multivariate AZTI Marine Biotic Index (MAMBI) |  |
|  | Spain (NEA) | Benthic Opportunistic Annelida/amphipods index (BO2A) | Andalusia region |
|  | Sweden (Baltic) | Swedish multimetric biological quality index (soft sediment infauna) (BQI-SE) |  |
|  | Sweden (NEA) | Swedish multimetric biological quality index (soft sediment infauna) (BQI-SE) |  |
|  | United Kingdom | Infaunal Quality Index (IQI) |  |
| Macroalgae and angiosperms | Bulgaria | Ecological index (EI) |  |
|  | Croatia | Cartography of Littoral and upper sublittoral rocky-shore communities (CARLIT) | Method using macroalgae |
|  | Croatia | Posidonia oceanica Multivariate Index (POMI) | Method using angiosperms |
|  | Cyprus | Ecological Evaluation Index (EEI-c) | Method using macroalgae |
|  | Cyprus | Posidonia oceanica Rapid Easy Index (PREMI) | Method using angiosperms |
|  | Denmark (Baltic) | Depth limit of Zostera marina (DepZos) |  |
|  | Estonia | Estonian coastal water phytobenthos Index (EPI) |  |
|  | Finland | Fucus depth limit (DepFuc) |  |
|  | France (MED) | Cartography of Littoral and uppersublittoral rocky-shore communities (CARLIT) | Method using macroalgae |
|  | France (MED) | Posidonia oceanica Rapid Easy Index (PREI) | Method using angiosperms |
|  | France (NEA) | Cover, Characteristic species, Opportunistic species on intertidal rocky bottoms (CCO) | Method using macroalgae |
|  | France (NEA) | Macroalgal Bloom Assessment (CWOGA)  Seagrass beds quality in coastal and | Method using opportunistic macroalgae |
|  | France (NEA) | Seagrass beds quality in coastal and transitional water bodies (SBQ) | Method using seagrasses |
|  | Germany (Baltic) | Baltic ALgae COmmunity AnalySIs System (Balcosis) | For the IC common types BC7 and BC8 |
|  | Germany (Baltic) | PHYtoBenthic Index for Baltic inner COastal waters (PHYBIBCO) | For the IC common type BC2 |
|  | Germany (NEA) | Assessment tool for intertidal seagrass in coastal and transitional waters (SG-DE) | Method using seagrasses |
|  | Germany (NEA) | Assessment of saltmarsh vegetation in coastal and transitional waters (EM) | Method using saltmarshes |
|  | Germany (NEA) | Opportunistic Macroalgae-cover/acreage on soft sediment intertidal in coastal waters (OMAI) | Method using opportunistic macroalgae |
|  | Greece | Ecological Evaluation Index (EEI-c) | Method using macroalgae |
|  | Greece | Cymo Skew | Method using angiosperms |
|  | Ireland | Opportunistic Green Macroalgal Abundance (OGA tool) | Method using opportunistic macroalgae |
|  | Ireland | Rocky Shore Reduced Species List (RSL-RS)  Intertidal Seagrass tool (SG-IE) | Method using seagrasses |
|  | Ireland | Saltmarsh Angiosperm Assessment Tool for Ireland (SMAATIE) | Method using saltmarshes |
|  | Italy | Cartography of Littoral and upper sublittoral rocky-shore communities (CARLIT) | Method using macroalgae |
|  | Italy | Posidonia oceanica Rapid Easy Index (PREI) | Method using angiosperms |
|  | Latvia | Maximum depth of the redalga Furcellaria lumbricalis distribution (MDFLD) | Method used for the IC common type BC5 |
|  | Latvia | Phytobenthos Ecological Quality Index (PEQI) | Method used fot the IC common type BC4 |
|  | Lithuania | Maximum depth of the redalga Furcellaria lumbricalis distribution (MDFLD) |  |
|  | Malta | Cartography of Littoral and uppersublittoral rocky-shore communities (CARLIT) | Method using macroalgae |
|  | Malta | Posidonia oceanica Rapid Easy Index (PREI) | Method using angiosperms |
|  | Netherlands | Monitoring beds of SG per waterbody using arial photographs, ground truth and specifying surface & density per species (SG-NL) | Method using seagrasses |
|  | Netherlands | WFD-metrics for natural watertypes: tidal salt marsh (TSM) | Method using saltmarshes |
|  | Norway | Multi Species Maximum Depth Index (MSMDI) | Method using subtidal macroalgae |
|  | Norway | Rocky Shore Reduced Species List with Abundance (RSLA) | Method using intertidal macroalgae |
|  | Poland | Macrophyte Quality Index (MaQI) |  |
|  | Portugal | Marine Macroalgae Assessment Tool (PMarMAT) | Method using macroalgae |
|  | Portugal | Seagrass quality index (SQI) | Method using seagrasses |
|  | Romania | Ecological index (EI) |  |
|  | Slovenia | Ecological Evaluation Index (EEI-c) |  |
|  | Spain (MED) | Cartography of Littoral and uppersublittoral rocky-shore communities (CARLIT) | Method using macroalgae |
|  | Spain (MED) | Posidonia oceanica Multivariate Index (POMI) | Method using angiosperms |
|  | Spain (MED) | Valencian classification system (Valencian-CS) | Method using angiosperms in Valencia and Murcia regions |
|  | Spain (NEA) | Rocky Intertidal Community Quality Index (RICQI) | Method used in Basque country |
|  | Spain (NEA) | Quality of Rocky Bottoms (CFR) | Method used in Cantabria region |
|  | Spain (NEA) | Reduced species list (RSL) | Method used in Andalusia region |
|  | United Kingdom | Opportunistic Macroalgae Blooming Tool (OMBT) | Method using opportunistic macroalgae |
|  | United Kingdom | Rocky Shore Reduced Species List (RSL-RS) | Method using macroalgae |
|  | United Kingdom | UK Saltmarsh Tool (SM) | Method using saltmarshes |
|  | United Kingdom | Intertidal Seagrass tool (SG-UK) | Method using segrasses |
